# South African health professional associations urged to end commercial milk formula industry sponsorship

**DOI:** 10.4102/jcmsa.v3i1.150

**Published:** 2025-04-04

**Authors:** Catherine J. Pereira-Kotze, Lori Lake, Max Kroon, Haroon Saloojee, Lisanne du Plessis, Zandile Kubeka, Sheila Clow, Renier Coetzee, Mariatha Yazbek, Nomajoni Ntombela, Sithembile Dlamini-Nqeketo, Gilbert Tshitaudzi, Silingene J. Ngcobo, Nthuseni S. Murudi-Manganye, Sue Fawcus, Nzama Mbalati, Tanya Doherty

**Affiliations:** 1 Health Systems Research Unit, South African Medical Research Council, Cape Town, South Africa; 2 Children’s Institute, Faculty of Health Sciences, University of Cape Town, Cape Town, South Africa; 3 Department of Neonatology, Faculty of Health Sciences, University of Cape Town, Cape Town, South Africa; 4 Mowbray Maternity Hospital, Cape Town, South Africa; 5 Department of Paediatrics and Child Health, Faculty of Health Sciences, University of the Witwatersrand, Johannesburg, South Africa; 6 Division of Human Nutrition, Global Health Department, Faculty of Medicine and Health Sciences, Stellenbosch University, Cape Town, South Africa; 7 Cluster: Child, Youth and School Health, National Department of Health, Pretoria, South Africa; 8 Division of Nursing and Midwifery, Department of Health and Rehabilitation Sciences, Faculty of Health Sciences, University of Cape Town, Cape Town, South Africa; 9 School of Public Health, Faculty of Community and Health Sciences, University of the Western Cape, Cape Town, South Africa; 10 Department of Nursing Science, Faculty of Health Sciences, University of Pretoria, Pretoria, South Africa; 11 International Baby Food Action Network (IBFAN) Africa, Pretoria, South Africa; 12 World Health Organization, Pretoria, South Africa; 13 United Nations Children’s Fund (UNICEF), Pretoria, South Africa; 14 Department of Nursing and Midwifery, College of Health Sciences, University of KwaZulu-Natal, Pietermaritzburg, South Africa; 15 School of Health Care Sciences, Department of Nursing Science, University of Pretoria, Pretoria, South Africa; 16 Department of Obstetrics and Gynaecology, Faculty of Health Sciences, University of Cape Town, Cape Town, South Africa; 17 Healthy Living Alliance (HEALA), Johannesburg, South Africa; 18 Department of Paediatrics and Child Health, Faculty of Health Sciences, University of Cape Town, Cape Town, South Africa

**Keywords:** conflicts of interest, health professionals, professional associations, sponsorship, financial incentive, breastmilk substitutes, commercial milk formula, breastfeeding, infant and young child nutrition

## Abstract

Despite the wealth of evidence about the benefits of breastfeeding, the commercial milk formula (CMF) industry continues to grow and project future profits, fuelled by aggressive and pervasive marketing practices that include the targeting of health care professionals. When health professionals and their associations accept funding from the CMF industry, this creates a conflict of interest (COI). The World Health Organization (WHO) and United Nations Children’s Fund (UNICEF) have therefore issued clear unequivocal guidance including through World Health Assembly resolutions that health care institutions and professional associations should refuse sponsorship from the CMF industry. We urge health professionals in South Africa to heed this call to protect their professional integrity and ensure that care and support for pregnant and breastfeeding women, children and families is free from commercial influence.

## Introduction

The benefits of breastfeeding are unequivocal and include public health, societal, economic and environmental advantages across the lifespan.^1^ Despite this, the commercial milk formula (CMF) industry continues to grow.^2^ The pervasive marketing practices used by the CMF industry have been well-documented and include active targeting of health professionals^3,4^ including doctors, nurses, midwives and dietitians, who are the trusted source of advice for parents on infant and young child feeding (IYCF). When health professionals and their associations accept funding from the CMF industry, this creates a conflict of interest (COI) and subconscious obligation, potentially influencing health professionals towards the industry’s commercial interests and marketing.^5^ There are guidelines on how to avoid such conflicts, yet the CMF industry continues to subvert global and national regulations.

## Global guidance on sponsorship of health professional associations

Efforts to protect the public (especially pregnant women, mothers, infants and young children) from the CMF industry date back to 1981 and the establishment of the International Code of Marketing of Breastmilk Substitutes and subsequent World Health Assembly (WHA) resolutions (hereafter referred to as ‘the Code’) designed to strengthen the regulatory framework and address emerging challenges such as COI and digital marketing.^6^ The Code aims to contribute to ensuring safe and adequate nutrition for infants by protecting and promoting breastfeeding, ensuring the proper use of CMF when necessary and enabling the IYCF environment to remain free from commercial influence.^6^ In addition, the World Health Organization (WHO) and United Nations Children’s Fund (UNICEF) have issued clear guidance (Box 1) calling on health professional associations ‘to refuse to accept sponsorship, in any form, from companies that market foods for infants and young children, including formula milk for children up to 36 months …’. This directive is relevant to all health care professionals and associations, whether working in public or private capacities, clinical practice, academia or public health domains. Recent UNICEF guidance to counter industry opposition to Code implementation reinforces that collaboration between the baby food industry and any part of the health system creates COI that cannot be managed and should therefore be avoided altogether.^7^

BOX 1 Summary of global guidance on sponsorship of healthcare professionals and their associations by the commercial milk formula industry.

**Guidance on ending the inappropriate promotion of foods for infants and young children** ^8^

**Recommendation 6.** ‘Companies that market foods for infants and young children should not create conflicts of interest in health facilities or throughout health systems. Health workers, health systems, health professional associations and nongovernmental organisations should likewise avoid such conflicts of interest. Such companies, or their representatives, should not:

▪ provide free products, samples or reduced-price foods for infants or young children to families through health workers or health facilities, except: as supplies distributed through officially sanctioned health programmes. Products distributed in such programmes should not display company brands
▪ donate or distribute equipment or services to health facilities
▪ give gifts or incentives to health care staff
▪ use health facilities to host events, contests or campaigns
▪ give any gifts or coupons to parents, caregivers and families
▪ directly or indirectly provide education to parents and other caregivers on infant and young child feeding in health facilities;
▪ provide any information for health workers other than that which is scientific and factual;
▪ sponsor meetings of health professionals and scientific meetings’.

**Clarification on sponsorship of health professional and scientific meetings by companies that market foods for infants and young children: Information note** ^9^

‘The WHO *Guidance on Ending the Inappropriate Promotion of Foods for Infants and Young Children* calls on governments to end sponsorship of meetings of health professionals and scientific meetings by companies that market foods for infants and young children, including formula milk for children up to 36 months. Therefore, companies that market these products should not be allowed to sponsor health professional or scientific meetings or events, regardless of what other products that company may also market. Professional and scientific associations or organisations who organise or host these events (including their representatives) should neither solicit nor accept sponsorship from companies that market foods for infants and children, including formula milks for children up to 36 months’.

‘In addition to providing funds to support the general operation of a meeting, conference or educational event, the activities listed below are also examples of sponsorship:

▪ Provision of in-kind support for specific activities of a conference
▪ Advertisements of any company, brand or product
▪ Sponsorship of sessions or side-sessions
▪ Financial support or aid, scholarships, awards or grants
▪ Sale of meeting delegates’ contact details
▪ Exhibition space’

**Sponsorship of health professional associations by manufacturers and distributors of commercial milk formula: technical documents** ^10,11,12^

In September 2024, the WHO published technical support for previous guidance that companies that market foods for infants and young children should not ‘sponsor meetings of health professionals and scientific meetings’. Three documents were published:

▪ Model policy
▪ Alternative funding resources for events
▪ Case studies from six countries (India, Italy, Australia/New Zealand, Ghana, United Kingdom and South Africa).

*Sources*: World Health Assembly (WHA). A69/7 Add. 1 Maternal, infant and young child nutrition – Report by the Secretariat: Guidance on ending the inappropriate promotion of foods for infants and young children [homepage on the Internet]. Geneva: WHO; 2016. Available from: https://apps.who.int/gb/ebwha/pdf_files/WHA69/A69_7Add1-en.pdf; WHO, UNICEF. Clarification on sponsorship of health professional and scientific meetings by companies that market foods for infants and young children [homepage on the Internet]. 2023. Available from: https://www.who.int/publications/i/item/9789240074422; World Health Organization (WHO). Sponsorship of health professional associations by manufacturers and distributors of commercial milk formula: Model policy [homepage on the Internet]. 2024. Available from: https://www.who.int/publications/i/item/B09120; World Health Organization. Sponsorship of health professional associations by manufacturers and distributors of commercial milk formula: Alternative funding resources for events. 2024; World Health Organization. Sponsorship of health professional associations by manufacturers and distributors of commercial milk formula: Case studies. 2024

Note: Please see full reference list of this article: https://doi.org/10.4102/jcmsa.v3i1.150.

WHO, World Health Organization.

## Why is the influence of the commercial milk formula industry on health professional associations a problem?

The commercial determinants of health refer to private sector and commercial activities that influence population health, often driven by industries and corporations.^13^ The products and practices of some of the largest and most powerful transnational corporations are causing illness, environmental harm, social and health inequity, highlighting the complex and interactive nature of commercial influences on health.^13^ It is estimated that just four industry sectors (tobacco, alcohol, ultra-processed food, fossil fuel) account for at least one-third of global deaths.^13^

Pregnant women, mothers, infants and young children present a novel case for the commercial determinants of health because of the vulnerability of these groups and the time-critical, life course impact of nutrition in the first 1000 days of life. The global CMF industry is highly profitable generating over $187 billion in revenue in 2020 with profit margins over 20%.^2^ Its primary interest is to increase sales and profits for shareholders, in direct opposition to the ethical responsibilities of health professionals to improve the health and well-being of patients and populations. Therefore, when the CMF industry provides financial incentives, such transactions can threaten the autonomy and integrity of health professionals and their associations, potentially leading them to intentionally or inadvertently prefer or recommend CMF, or prescribing specialised CMF when these are unnecessary.^14^ One clear example of this was the overdiagnosis of cow’s milk allergy, and 500% increase in prescriptions of specialist formula milk in the United Kingdom (UK) between 2006 and 2016, with no evidence for such a large increase in true prevalence. This overdiagnosis was driven by the CMF industry through extensive links between industry and cow’s milk allergy research, guideline development, medical and public education.^14^

Despite claims that they are committed to responsible marketing, public health and sustainable development, CMF industry practices often do not align with their brand promises. There are many examples of repeated violations of the Code and national legislation, globally and nationally.^4,15^ The CMF industry has unconscionably used global health epidemics (like human immunodeficiency viruses [HIV]) and pandemics (like coronavirus disease 2019 [COVID-19]) for marketing purposes, by creating fear around disease transmission through breastmilk.^16,17,18^ It has recently been exposed that Nestle adds higher levels of sugar to baby food products sold in low- and middle-income countries (LMICs) compared to products sold in high-income countries.^19^ This can only be described as discriminatory and predatory, strengthening the stance that health professionals should not associate themselves with these companies in any way.

There are limited instances when CMF may be necessary for medical reasons, or in situations where the caregiver is not the child’s biological parent and donor human milk is not available. Women and parents are also free to choose how to feed their children, but it is important that these choices are free from commercial pressures. For most mothers and infants where breastfeeding is possible, improving breastfeeding to near universal levels could prevent 823 000 deaths of children under 5 years and improve long-term health outcomes of growth restricted infants into late adulthood. Furthermore, improved breastfeeding could prevent up to 98 243 deaths among women from breast cancer, ovarian cancer and diabetes, annually.^20^ In direct contrast, CMF feeding is associated with risks of contamination, in both high-income contexts and LMIC, and more so when sanitation and the economic means for safe preparation and sustained supply are uncertain.^21^ The production and preparation of CMF, including emissions from its transport, production and in-home sterilisation of bottles, contribute to greenhouse gas emissions and increased usage and waste of water and plastic, resulting in CMF use having a considerably larger carbon footprint than breastfeeding.^22^

## Infant and young child feeding and health in South Africa

In the context of pervasive CMF marketing, despite increased breastfeeding, rates remain suboptimal in South Africa (SA) and globally.^23^ Exclusive breastfeeding (EBF) under 6 months in SA was 32% in 2016^24^ but is estimated at 22% in 2022.^25^ South Africa is therefore far off track to reach the global EBF target of 70% by 2030. The SA National Department of Health monitors the prevalence of EBF at 14 weeks and this has decreased from 47.8% in 2017/2018 to 44.6% in 2021/2022.^26^

In 2020, 60% of children aged 0–4 years were categorised as experiencing multi-dimensional poverty (i.e. suffering from four out of seven deprivations including water, sanitation and hygiene) in SA, contributing to the use of CMF being associated with increased risk for diarrhoea and malnutrition, both leading causes of under-five mortality in SA.^27^ Use of CMF in preterm babies is associated with increased risk and severity of necrotising enterocolitis and mortality compared to human milk.^28^ In these babies, supplementation of own mothers’ milk with donated human milk rather than preterm formula is cost-effective, decreases incidence of necrotising enterocolitis and reduces length of hospital stays and post-discharge costs of medical care.^29^ Recently, a United States court ordered Abbott Laboratories to pay almost $500 million for failing to adequately warn consumers of potential risks of using a specialised formula for preterm infants.^30^

## South African Regulations

In 2012, SA legislated the *Regulations Relating to Foodstuffs for Infants and Young Children* (R991), housed within the *Foodstuffs, Cosmetics and Disinfectants Act* (54 of 1972), to legalise and enforce the Code.^31^ While R991 prohibits health professionals working with infants and young children from accepting any direct financial contributions or sponsorship from CMF manufacturers, they currently allow sponsorship of meetings for health professionals where infant and young child nutrition is on the agenda, provided it is paid into a pool of funds in a fair and transparent manner. The current regulations do not prohibit industry from offering financial contributions to professional associations or their events and are being revised to address these loopholes and ensure alignment with latest evidence and global guidance.

## Commercial milk formula marketing and health professional associations in South Africa

The CMF industry uses many marketing strategies, and one route of influence is through financial support of health professionals and their associations. This includes using health professionals as ‘category entry points’ for the marketing of CMF and there is evidence of the CMF industry attempting to influence science academia, public policy and clinical guidelines.^3^ Data collected during 2020/2021 in SA confirmed that health professionals, particularly those working in the private sector, are exposed to, and used as, conduits to promote CMF use among mothers^32^ (Box 2). This is carried out through industry representatives frequently visiting health professionals to present products, provide training or sponsor educational activities featuring their company’s products,^32^ and in turn, health professionals are more likely to recommend specific companies and brands (Box 2). Representatives of CMF manufacturers have a history of providing health professionals with company-branded gifts and samples, and even though this is prohibited by R991 in SA, the practice still takes place.^33^ Health professionals should not publicly endorse CMF products, and any engagement with the CMF industry and its representatives should be limited to scientific and factual matters. Companies are also pathologising normal infant behaviour (such as posseting, colic, flatulence, constipation and nighttime waking) and capitalising on gaps in health professional training to further market ‘specialised’ formula milks to – and through – health professionals.^3^ All health professionals should receive appropriate undergraduate and in-service training on COI, the Code and national legislation to implement the Code.

BOX 2 Examples of responses from and explanations by health professionals and parents in South Africa regarding their experience with commercial milk formula marketing.

- Description provided by a general practitioner working in private practice in Cape Town, SA:
  ▪ ‘I see the formula reps a lot. I see at least 3 of them that I see regularly. They tell me all the latest and I can never remember all of the special things that they tell me. I have millions of pamphlets. Yeah, and they push their products through me’.^32^
- Feedback from a Doula working at a private clinic in Johannesburg, SA:
  ▪ ‘Brand X is being sponsored to the hospital. If it doesn’t work, we will recommend another one within the brand X range. I trust the brand’.^34^
- Explanation provided by mothers participating in focus group discussions with women who were mixed feeding in SA:
  ▪ ‘Actually, for me it was a doctor who told me that I should use Pelargon as it is light and therefore similar to breast milk. The doctor was actually saying even if the mom is not breastfeeding the child, if they are giving them Pelargon it’s actually tantamount to giving them breast milk’.^35^
  ▪ ‘Well, my paediatrician only spoke Nestle products so I’ve only known Nan and Lactogen, so with my son now I did the same start process, he was on the allergy and then on Nan for two months and then Lactogen. I switched to Lactogen because it’s cheaper, it’s like R100 cheaper than what Nan is and it’s the same product, same company’.^35^

*Source*: Doherty T, Pereira-Kotze CJ, Luthuli S, et al. They push their products through me: health professionals’ perspectives on and exposure to marketing of commercial milk formula in Cape Town and Johannesburg, South Africa – A qualitative study. BMJ Open. 2022;12(4):e055872; WHO, UNICEF. How the marketing of formula milk influences our decisions on infant feeding: Report – South Africa [homepage on the Internet]. 2022. Available from: https://www.who.int/publications/i/item/9789240055216; Horwood C, Luthuli S, Pereira-Kotze C, et al. An exploration of pregnant women and mothers’ attitudes, perceptions and experiences of formula feeding and formula marketing, and the factors that influence decision-making about infant feeding in South Africa. BMC Public Health. 2022;22:393

Note: Please see full reference list of this article: https://doi.org/10.4102/jcmsa.v3i1.150.

In SA, the CMF industry targets specific health professionals and their associations, in the public and private sectors, academics and scientists. Some organisations have put measures in place to curb sponsorship, while other partnerships continue to grow. In 2019, the Department of Paediatrics and Child Health (DPCH) at the University of Cape Town (UCT) developed a position statement (https://health.uct.ac.za/sites/default/files/content_migration/health_uct_ac_za/547/files/UCT_Department_of_Paediatrics_position_statement_on_Relations_with_Formula_Milk_Companies-13November2019.pdf) that ended any further sponsorship from the CMF industry. The paediatric department at the University of the Witwatersrand previously accepted Nestle sponsorship of an award to the highest achieving final year medical student in the discipline of Paediatrics (https://www.wits.ac.za/media/wits-university/students/graduations/2022-graduations/14%20Dec%2014-30%20December_38.pdf#page=23) but has decisively severed ties with Nestle’s sponsorship, withdrawing from this arrangement. The United South African Neonatal Association (USANA) published a public position statement (https://usana.org.za/wp-content/uploads/USANA-Position-Statement-on-relations-with-Commercial-Milk-Formula-Companies_September-2022.pdf) in 2022 indicating that it will not accept financial support from CMF companies. Association of Dietetics in South Africa (ADSA) has a progressive sponsorship policy (https://adsa.org.za/wp-content/uploads/2023/05/ADSA-Sponsorship-Guidelines-December-2019.pdf), whereby they avoid funding from CMF manufacturers as well as companies manufacturing sugar sweetened beverages, fast-foods and most ultra-processed foods.

While the Allergy Foundation of South Africa (AFSA) has an existing partnership (https://www.allergyfoundation.co.za/about-afsa/) with the Nestle Nutrition Institute, the Allergy Society of South Africa (ALLSA) has published a public position (https://allsa.org/wp-content/uploads/2021/11/ALLSA-Position-statement-on-Relations-with-Formula-Milk-Companies.pdf) stating that they will not violate the International Code and national R991 regulations, and that any industry relationships would be monitored internally by an ethics and compliance committee established for this purpose. Yet this self-regulation has proved problematic and has allowed industry interference to continue.

We urge all health professional associations and academic institutions to follow the lead of organisations such as the UCT DPCH, USANA and ADSA, in setting a new standard for ethical conduct by publishing clear and strong public position statements aligned with global guidance. The College of Medicines of South Africa (CMSA) is the national examining body for all medical postgraduate specialist, sub-specialist and diploma training in South Africa, has a membership of over 11 000 people and is considered ‘the custodian of the quality of medical care in South Africa’. The CMSA is therefore well-positioned to take the lead among health professionals in South Africa in advocating for strong COI position statements and policies, especially as the profile of its membership (specialists) infers it is likely that many CMSA members are in senior positions, respected in their fields and workplaces and colleagues may follow their lead. We encourage the CMSA as the custodian of postgraduate medical education to develop and/or strengthen their own COI policy or adapt existing policies, to explicitly refer to ending sponsorship of health professional associations from CMF manufacturers, drawing on WHO guidance and following the lead of other associations. The *Journal of the Colleges of Medicine of South Africa* (JCMSA) aims to use its structure to develop aspects of medical and dental training and professionalism, which are common to all specialities (https://jcmsa.org.za/index.php/jcmsa/pages/view/journal-information/editorialTeamBio/5934).

In SA, CMF manufacturers continue to sponsor and provide continuing professional development (CPD) training (https://www.nestlenutritiononline.co.za/user/register; https://www.nutricia.co.za/login).^32^ In 2024, London et al., provided a strong justification that the provision of CPD training should be independent from vested interests^36^ and describe that ‘In 2024, the CMSA adopted a policy prohibiting acceptance of donations or support from health-harming industries’. Health professionals should be wary of organisations that offer CPD training that are, in reality, proxies for, and/or funded by, the CMF industry. Examples of this include training institutes within CMF companies (such as the Nestle Nutrition Institute), patient support groups (such as provided through AFSA) and CPD activities provided through industry funded associations (such as the Infant Feeding Association). It is important that the Health Professions Council of South Africa and other CPD accrediting bodies stop enabling the sponsorship of CPD training by CMF manufacturers. Regulation R991 that enacts the Code should also be revised to address this loophole (which allows CMF manufacturers to sponsor health professional events, if they contribute to a pool of funds) as part of broader efforts to strengthen SA legislation to bring it in line with the most recent WHA resolutions and global guidance.

## South African health professional associations need to end all sponsorship relationships with the commercial milk formula industry

In 2019, the UK Royal College of Paediatrics and Child Health declared they would stop accepting advertising or conference stands promoting CMF (https://www.rcpch.ac.uk/sites/default/files/2019-12/acceptance_and_refusal_of_donations_policy_v2_2019-12.pdf.pdf). Since then, there was a call by South African academics and health professionals for academic departments and professional associations in SA to adopt clear position statements to eliminate COI.^37^ An increasing number of organisations no longer accept sponsorship from the CMF industry. In March 2024, a group of six international health professional associations published a letter calling on all health professional associations to end sponsorship relationships with companies that market CMF by the end of 2024.^38^ We would like to amplify this call and appeal to SA health professional associations, regulatory bodies and academic institutions to make the same commitments. To improve our capacity to deliver quality care to all women, families and children, health professionals, their associations and regulatory bodies need to be free from commercial influence.

We call on all health professional associations in South Africa to abide by the guidance issued by the WHO and UNICEF and to adopt clear public position statements that refuse sponsorship in any form from the CMF industry.
